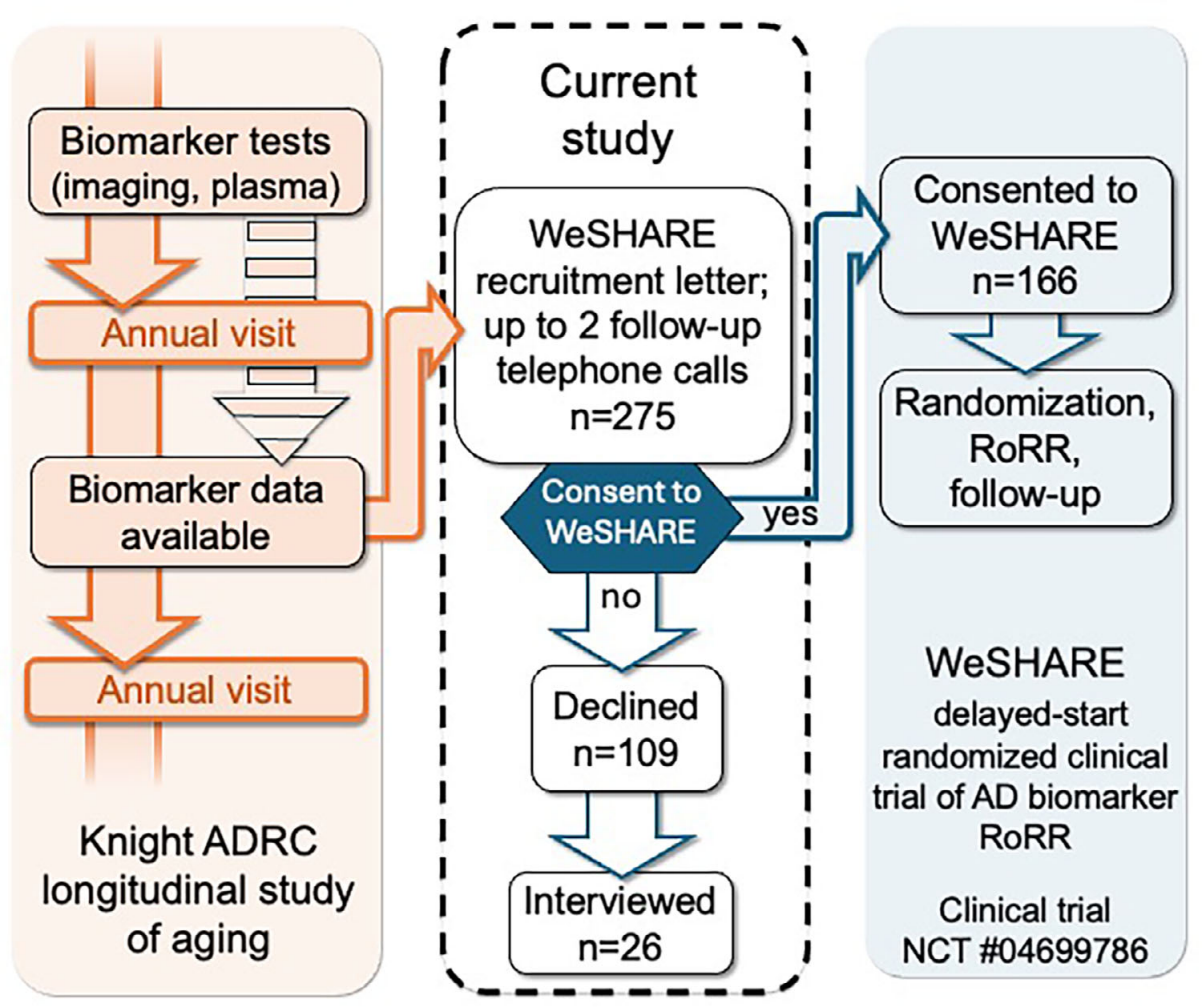# Declining to learn results of research biomarker tests for Alzheimer Disease

**DOI:** 10.1002/alz70858_101067

**Published:** 2025-12-25

**Authors:** Spondita Goswami, Sarah Hartz, Amy Oliver, Sacha Jackson, Tomi Ogungbenle, Alissa Evans, Erin Linnenbringer, Krista L. Moulder, John C. Morris, Jessica Mozersky

**Affiliations:** ^1^ Washington University in St Louis, St Louis, MO, USA; ^2^ Washington University in St. Louis, St. Louis, MO, USA; ^3^ St Louis University, St Louis, MO, USA; ^4^ The Charles F. and Joanne Knight Alzheimer Disease Research Center, St. Louis, MO, USA; ^5^ Washington University School of Medicine, St. Louis, MO, USA; ^6^ Washington University School of Medicine in St. Louis, St. Louis, MO, USA; ^7^ Knight Alzheimer Disease Research Center, St Louis, MO, USA; ^8^ Bioethics Research Center, St Louis, MO, USA

## Abstract

**Background:**

This is the first study to quantitively and qualitatively evaluate who declines to learn individual AD biomarker research results and what influences the decision.

**Method:**

Participants with no prior option to receive research results were offered the option to learn these results. 274 individuals aged ≥65 with unimpaired cognition enrolled in a longitudinal cohort of aging at the Knight Alzheimer Disease Research Center and available biomarker data [*APOE* genotype and either imaging (amyloid PET and MRI) or plasma amyloid] were approached. The primary outcome was the decision to receive AD research biomarker results. We evaluated whether this decision differed by demographic factors: race, parental history of AD, age, gender, and type of biomarker result offered (imaging or plasma). Semi‐structured qualitative interviews with a subset of participants who declined receiving research results explored reasons for declining.

**Results:**

274 participants [average age 75.9 (SD 5.8); 57% women; 13% self‐identified as Black] were offered their research results and 40% declined. Black participants were more likely to decline than White participants (adjusted RR=1.92, 95% CI 1.45‐2.53). Participants with a known parental history of AD dementia were more likely to decline than those without (adjusted RR=2.3, 95% CI 1.3‐4.3). Qualitative interviews found the following reasons for declining: knowing would be a burden, negative experiences and perceptions of AD dementia, feeling good about memory currently, familial burden, already being prepared, and the uncertainty of results.

**Conclusion:**

In this study of participants enrolled in a longitudinal cohort of aging offered their research results, those with a parental history of AD dementia and Black participants were significantly more likely to decline. Qualitative interviews suggest that a family history of AD can create negative experiences and perceptions of the disease, which may influence the decision to learn results. Further research is needed to better understand racial differences in uptake and ensure that the choice to receive research results reflect individual preferences and wishes.